# Effect of different bleaching protocols on whitening efficiency and enamel superficial microhardness

**DOI:** 10.4317/jced.54967

**Published:** 2018-08-01

**Authors:** Amanda-Mahammad Mushashe, Beatriz-Serrato Coelho, Paula-Pontes Garcia, Bruna-Cristina-do Nascimento Rechia, Leonardo-Fernandes da Cunha, Gisele-Maria Correr, Carla-Castiglia Gonzaga

**Affiliations:** 1 DDS, MSc, Graduate student of the Graduate Program in Dentistry, Universidade Positivo, Curitiba, PR, Brazil; 2 DDS, MSc, PhD, Professor of the Graduate Program in Dentistry, Universidade Positivo, Curitiba, PR, Brazil

## Abstract

**Background:**

Tooth bleaching is a popular aesthetic treatment to modify the color of teeth. Despite the extensive literature concerning the subject, there is still no consensus regarding the application mode of the different bleaching agents and their effect on enamel. Therefore, this study evaluated the influence of different bleaching protocols on whitening efficiency and enamel superficial hardness.

**Material and Methods:**

Bovine enamel fragments were embedded in acrylic resin and wet-sanded to obtain a flat buccal surface. The specimens were then randomly divided into 6 groups (n=10), based on the bleaching material [HP Maxx 35% (35% hydrogen peroxide), HP Blue 35% (35% hydrogen peroxide + Ca) and Whiteness Perfect 10% (10% carbamide peroxide)] and application mode (3 applications of 15 min, 1 application of 45 min, 1 application of 1h30 or 1 application of 3h30). The color and superficial hardness were assessed before and after bleaching. The color was assessed by means of a digital spectrophotometer, using CIELab parameters. Vickers hardness was determined using a load of 200g for 10s. Data were statistically analyzed by one-way ANOVA with repeated measures and Tukey’s test (α = 0.05).

**Results:**

Concerning the color alteration, the groups were similar to each other, except for at-home bleaching protocols, which were statistically different from each other. Similarly, there was a reduction in hardness values comparing the initial and final periods, with lowest final hardness for the at-home bleaching protocol group applied for 3h30.

**Conclusions:**

It can be concluded that the bleaching protocols influenced the final color and enamel surface hardness, evidencing that lower gel concentrations applied for longer periods promoted greater variation in color and lower final hardness.

** Key words:**Tooth bleaching, roughness, microhardness, in-office, at-home bleaching.

## Introduction

Tooth bleaching is a popular aesthetic treatment to modify the color of teeth. In-office, at-home or the association of both techniques can be used for vital tooth whitening (1). Despite the extensive literature concerning the subject, there is still no consensus regarding the application mode of the different bleaching agents and their effect on enamel. The efficiency and effects of such protocols must be assessed to enhance the clinical practice (2,3).

The success of bleaching protocols is directly related to the diffusion capacity of the hydrogen peroxide. This gel can be applied directly to the tooth, or may be produced locally in a chemical reaction, having as precursors the sodium perborate or the carbamide peroxide. The action mechanism of peroxide is through oxidizing the organic matrix within the substrate, making the tooth whiter and more opaque. Highly concentrated hydrogen peroxide whitening gels used during in-office tooth whitening can be left on the tooth surface for nearly 15 minutes and replenished 2 or 3 times. Lower concentrations of gel, such as the ones used for the at-home technique, are used for longer periods of time (4). Regarding gel concentration, the presence of greater number of reactive molecules generally leads to increased activity, although the response at higher concentrations is not necessarily linear for whitening products (5). Thus, it can be considered that tooth bleaching is concentration- and time-dependent.

As the oxidation reaction occurs, after the gel is applied on the tooth surface, there is degradation of the peroxide. Over time, the pH would become acidic, decreasing the effectiveness of the gel action and possibly damaging the tooth structure. The decrease of enamel hardness after bleaching procedures, as an indicator for mineral loss (6), would support the rationale for replenishing the whitening gel during in-office techniques.

Therefore, the aim of this study was to evaluate the effects on whitening efficiency and enamel hardness of different bleaching protocols. The null hypothesis tested was that bleaching protocol would not interfere in the color change or the enamel microhardness.

## Material and Methods

Sixty bovine incisors, recently extracted, free of caries, crack or any enamel defects, were sectioned, cleaned and stored in 0.5% chloramine T, at 5ºC, until use. Enamel fragments (5 x 5 x 3 mm) were obtained using a low-speed diamond saw (Isomet 1000; Buehler, Lake Bluff, IL, USA) and then embedded in PVC cylinders using acrylic resin (Jet; Articles Dental Classic LTDA; São Paulo, SP, Brazil). After, samples were wet-sanded with 400- and 600- grit silicon carbide paper (Metaserv; Buehler, Lake Bluff, IL, USA), in order to expose a flat buccal surface, and placed under water in ultrasonic cleaner for 5 min. The specimens were then randomly divided into six groups (n = 10), according to the bleaching gel and application mode ( Table 1), and stored in distilled water.

**Table 1:**
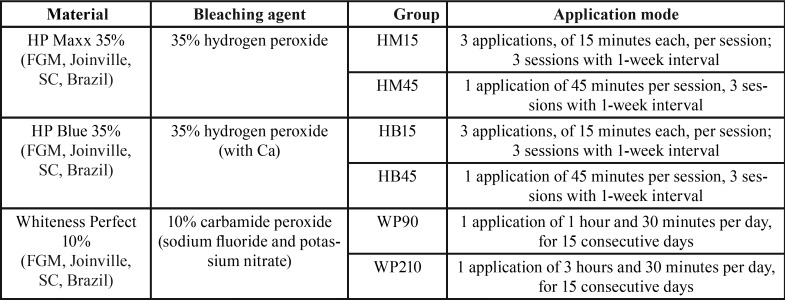
Bleaching gels and application modes.

After allocation, the specimens were submitted to respective treatments. Before the bleaching procedure, specimens were gently dried with cotton for moisture control. The bleaching gels were applied on the enamel surface using a spatula, obtaining a uniform gel layer of 2-mm thickness. After the gel’s application time, the specimens were abundantly rinsed with distilled water until complete removal of the product.

On the last session of each protocol, after treatment, the specimens were submitted to topical application of fluoride gel (Fluorgel, Nova DFL, Rio de Janeiro, RJ, Brazil) for 4 minutes, according to the manufacturer’s instructions, to induce the enamel remineralization process. During the whole experiment, the samples were maintained in distilled water at 37oC.

**Figure 1:**
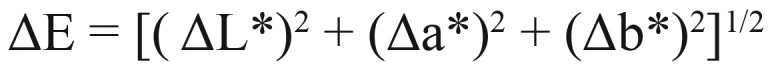
Equation.

The specimens were assessed as to color difference and enamel surface microhardness before the bleaching protocol (baseline) and after the remineralization process. Color measurement was performed using a digital spectrophotometer (Easyshade Advence, VITA Zahnfabrik, Bad Säckingen, Germany) under a standardized white background. The spectrophotometric data obtained for each specimen were recorded as CIELab parameters. The value L * represents the degree of luminosity of a specimen and varies from black (0) to white (100). The values a* and b* represent the degree of red (+a) - green (-a) and yellow (+b) - blue (-b) in the specimen, respectively. Total color difference (ΔE) was calculated by the following equation, (Fig. 1):

where ΔL*, Δa* and Δb* are the differences between the parameters before and after bleaching.

The microhardness was measured using a Vickers testing machine, under a vertical load of 200 g for 10 s. Three indentations were performed on the enamel surface and the mean microhardness values were recorded.

Color change and microhardness data were statistically analyzed by one-way ANOVA with repeated measures and Tukey’s test. All analyzes were performed with a significance level of 5%.

## Results

The means and standard deviations for color change according to the groups are described in  Table 2. Statistical analysis showed significant differences for the groups evaluated (*p* = 0.0124). The groups of at-home bleaching protocol were statistically different from each other, and specimens in the WP210 group showed greater color variation. Groups HM15 and WP90 presented the least variations, being statistically similar to groups HM45 and HB15.

**Table 2:**
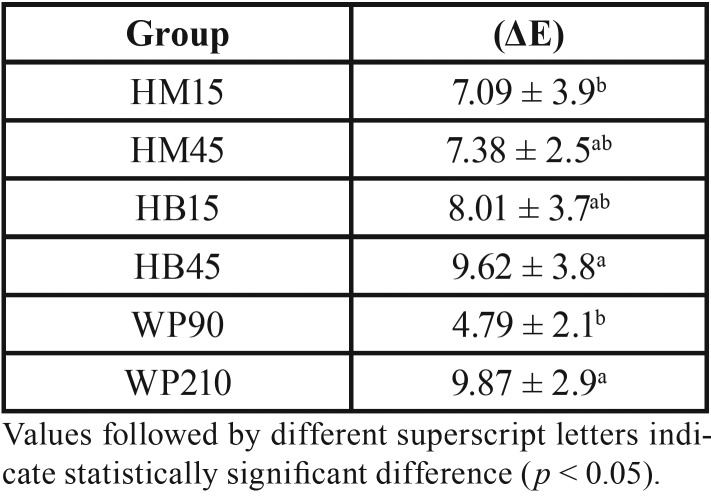
Means and standard deviations (SD) for color change (ΔE) in the different groups (n = 10).

The means and standard deviations of microhardness results before and after the bleaching procedure are described in  Table 3. Statistical analysis showed significant differences for the individual factors groups (*p* = 0.0002) and time (*p* = 0.0488), and double interaction (*p* = 0.0016).

**Table 3:**
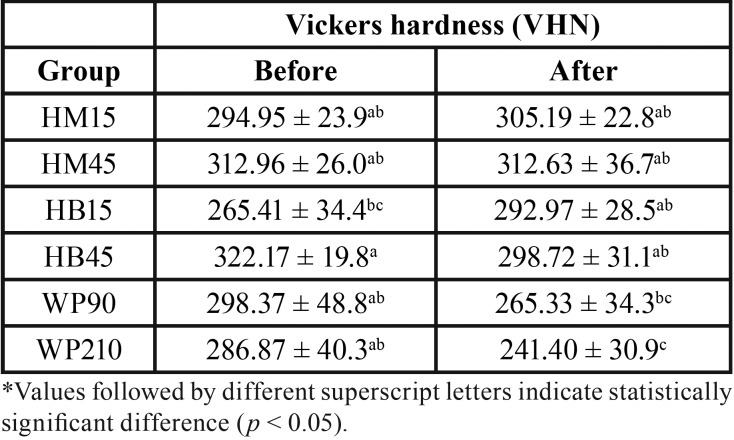
Means and standard deviations for the Vickers hardness before and after bleaching in the different groups (n = 10).

For the bleaching gels and protocols, the hardness values varied in the following order: HM45 (312.80)a < HB45 (310.45)ab < HM15 (300.08)ab < WP90 (281.85)abc < HB15 (279.19)bc < WP120 (264.14)c. The WP210 group showed the lowest hardness values (264.14), being statistically different from groups of in-office bleaching protocol. The other groups showed similar hardness values. Regarding the bleaching gel, HM45 group presented the highest result, presenting similar outcome as the WP90, HM15 and HB45 groups. WP210 presented the lowest results, being similar to groups HB15 and WP90. As for time, the initial hardness (296.79) was higher than the final hardness (286.04).

## Discussion

This study assessed the effectiveness of different whitening agents and their effect on enamel microhardness. Color change can be evaluated visually or by instrumental techniques. While visual guides can be considered as subjective analysis, measurement with spectrophotometers can be thought of as an objective method. In this study it was used a digital spectrophotometer, which is capable of detecting color differences below the threshold of visual perception (7). Furthermore, microhardness test is a normally used method to establish the mechanical properties of enamel after bleaching procedures, being that these measurements might be demonstrative for the mineral content of the bleached specimens (8).

According to this study, all bleaching protocols tested, both at-home and in-office, were effective (ΔE > 4), corroborating the results of Bernardon *et al.* (9). This emphasizes the effectiveness of bleaching agents based on peroxide hydrogen or carbamide peroxide, regardless of the concentration or application protocol. The present outcomes indicated that replenishing the gel at every 15 min for 3 times at the same section (as recommended by some manufacturers) did not affect the efficiency of treatment. Al-Harbi *et al.* (10) also observed no significant difference in the effectiveness of treatment performing 2 applications of 30 min or 4 of 15 min. This is an advantage, since less material is consumed, and the dentist does not need to change the material, thus also demanding less time for each session.

The penetration of bleaching agents through the dental tissues may be relevant regarding its effect on the mechanical properties of both enamel and dentin. However, studies (11-13) have shown that common bleaching protocols did not affect some crucial dentin properties, such as permeability and flexure strength, leading one to consider that the enamel tissue may be more affected by the respective agents. Therefore, this study assessed only the enamel microhardness, considering it is commonly the first substrate in contact with the bleaching gels.

It has been demonstrated that the adverse effects of bleaching products on enamel may depend on the type of bleaching agent or the concentration of hydrogen peroxide and duration of bleaching (14,15). In the present study, both at-home and in-office bleaching did not result in significant decrease in enamel microhardness, which partially accepts the null hypothesis. The evidence showed by this study agrees with those of Fatima *et al.* (16). The authors tested bleaching with 38% hydrogen peroxide and 16% carbamide peroxide, which also resulted in insignificant effect on enamel surface microhardness.

Studies have reported that adding calcium to bleaching agents prevents changes in enamel hardness and morphology without reducing the bleaching efficacy, in vitro (17). The literature and the manufacturer of bleaching gels with calcium suggest that this ion is intended to minimize the enamel demineralization process. However, in-office products tested in this study with (HP Blue) and without (HP Maxx) calcium were not significantly different regarding the microhardness. Only the long application of carbamide peroxide presented significant difference. Similar results were presented by Parreiras *et al.* (18).

The prolonged application of peroxide without minimizing the color changes of the bleaching treatment or significantly decreasing enamel microhardness could be clinically relevant, once less material would be needed and the procedure would be less time-consuming. However, tooth sensitivity may be intensified when prolonged application time of hydrogen peroxide is performed (19). Therefore, more studies regarding the cytotoxicity of such protocols must be performed.

Within the limitations of this study, it can be concluded that the bleaching protocols influenced the final color and enamel surface hardness, evidencing that lower gel concentrations applied for longer periods promoted greater variation in color and lower final hardness. Bleaching gels applied for long periods may be an interesting alternative to obtain effective whitening without significantly reducing the enamel hardness.
